# Melatonin-induced symptomatic bradycardia in an otherwise healthy male: a case report

**DOI:** 10.1093/omcr/omae096

**Published:** 2024-08-26

**Authors:** Asim Alawad, Wala Sati, Sara M I Ahmed, Moayed Elgassim, Mohamad Elgassim, Abderahman Balal

**Affiliations:** Emergency Medicine Department, Hamad General Hospital, P.O. Box: 3050, Doha, Qatar; Emergency Medicine Department, Hamad General Hospital, P.O. Box: 3050, Doha, Qatar; Emergency Medicine Department, Hamad General Hospital, P.O. Box: 3050, Doha, Qatar; Emergency Medicine Department, Hamad General Hospital, P.O. Box: 3050, Doha, Qatar; Emergency Medicine Department, Hamad General Hospital, P.O. Box: 3050, Doha, Qatar; Emergency Medicine Department, Hamad General Hospital, P.O. Box: 3050, Doha, Qatar

**Keywords:** melatonin, bradycardia, melatonin side-effect

## Abstract

Melatonin, a pineal gland hormone closely associated with the circadian rhythm, has been trending over the past years as an over-the-counter medication to aid with sleep disturbances. Although generally believed to be safe, recent studies show negative inotropic and chronotropic effects on the heart rate and blood pressure in humans. Several studies suggested that melatonin induces cardiac vagal tone and affects heart rate and mean arterial pressure. Limited literature is currently available on the effects of melatonin beyond its sleep function. We present a case of a healthy 22-year-old male who visited the emergency department reporting palpitations and dizziness following the ingestion of 20 mg of melatonin. Subsequent examinations revealed marked bradycardia. Fortunately, the patient experienced spontaneous resolution of the bradycardia without necessitating intervention after a few hours of observation, and he was observed and discharged.

## Introduction

Melatonin is a methoxyindole compound synthesized and released mainly from the pineal gland under the influence of the suprachiasmatic nuclei in synchronicity with the circadian rhythm. Its primary action is to transmit signals about darkness with the inference of the circadian control cycle to body physiology [1]. A few decades ago, synthetic melatonin was introduced to the market and has been used as a supplement to aid with sleep disturbances. Despite it being considered a relatively safe drug, some studies have reported a few side effects involving the cardiovascular system, among others [2]. We present a case of a healthy adult male who exhibited cardiovascular symptoms and was subsequently diagnosed with melatonin-induced bradycardia after 20 mg of exogenous melatonin intake.

## Case presentation

We report a case of a previously healthy 22-year-old male who works as an officer in the military. He presented to the ED with complaints of palpitations and dizziness after taking 2 tablets of 10 mg (total dose of 20 mg) of melatonin. The patient denied consumption of any additional medications earlier or any possible co-ingestion. He reported mild chest discomfort at the time of the onset of the palpitations but denied associated shortness of breath or any previous history of palpitations or syncope. His symptoms began approximately 30 min after the ingestion.

**Figure 1:**
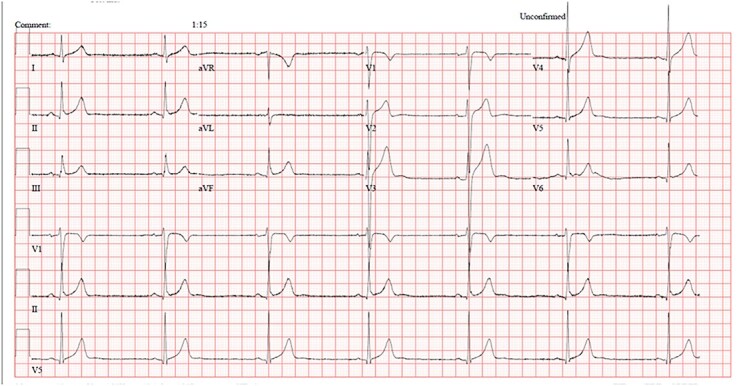
ECG showing sinus bradycardia with heart rate of 39, early repolarization and no other changes.

During assessment, the patient’s vitals recorded a heart rate (HR) of 38, a blood pressure reading of 128/72, and an oxygen saturation of 100%. His Glasgow Coma Scale (GCS) was 15/15, and he was ambulatory. His neurological assessment was within normal, with no signs of ataxia or slurred speech noted. His cardiac exam showed normal heart sounds without audible murmurs or added sounds. An electrocardiogram (ECG) was performed, demonstrating sinus bradycardia with a heart rate of 39 beats per minute, in conjunction with benign early repolarization. The intervals and QRS complexes manifested within normal parameters, and there were no discernible signs of ischemic changes or conduction delays (Fig. 1). Regrettably, there was no baseline ECG available for the patient, hindering reference comparison. Nonetheless, it was noted that the patient’s baseline HR usually ranges in the 50s and 70s but has never dropped to the 40s. Given the initial HR, the patient was placed on a cardiac monitor, and blood investigations, including his electrolyte panel and thyroid function tests, were sent.

The toxicology and cardiology teams were involved. However, the toxicology team deemed melatonin to be generally a safe drug and they recommended further monitoring while awaiting further cardiology evaluation and his test results. During the cardiologist’s assessment, an interesting pattern emerged in the patient’s HR variable response to position changes: supine, sitting, and standing. Sequentially, the recorded metrics were a blood pressure (BP) of 110/61 and HR of 57/min, a BP of 106/64 and HR of 75/min, and a BP of 124/67 and HR of 85/min, respectively, with no emergent new symptoms. Echocardiography revealed a normal left ventricular (LV) function, a biplane left ventricular ejection fraction (LVEF) of 60%, with no regional wall motion abnormalities, and a healthy right ventricle (RV). The comprehensive blood tests yielded results within normal ranges. Electrolyte levels were as follows: Sodium 141 mmol/l (normal range: 133–146), Potassium 3.8 mmol/l (normal range: 3.5–5.3), Chloride 105 mmol/l (normal range: 95–108), and Adjusted Calcium 2.27 mmol/l (normal range: 2.20–2.60). Additionally, Magnesium was measured at 0.78 mmol/l (normal range: 0.70–1.00). Thyroid function tests revealed TSH levels of 2.76 mIU/l (normal range: 0.30–4.20) and FT4 levels of 17.8 mmol/l (normal range: 11.0–23.3). Furthermore, two sets of Troponin-T levels, measured three hours apart, were recorded at 15 and 13 ng/l, respectively (normal range: 15–3).

**Figure 2:**
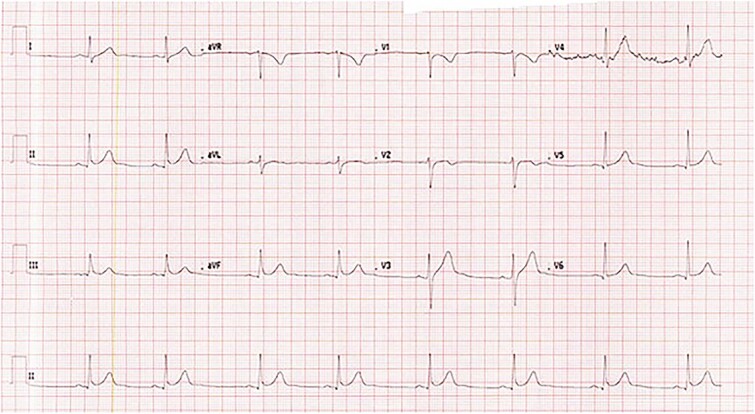
ECG showing sinus bradycardia with increased heart rate to 48.

Following four hours of close monitoring, the patient’s HR spontaneously increased to the 50s, and the patient remained asymptomatic throughout his hospital stay. The follow-up ECG few hours prior the time of discharge revealed a sinus bradycardia with no evident abnormalities (Fig. 2). Consequently, the patient was discharged in good health and was able to ambulate with a follow-up appointment scheduled with the cardiology team. According to the electrophysiology cardiologist, these ECG findings are indicative of a normal variant, considering that he is a young patient, no pathological findings in ECG, his normal echocardiography results and negative family history.

## Discussion

Melatonin, a hormone primarily secreted in a rhythmic pattern by the pineal gland, is particularly in young and middle-aged people. It’s notable that melatonin is also present in a concentration that far exceeds that of the pineal gland in extrapineal sites, such as the gastrointestinal tract (GIT). Due to its considerable size, the entire GIT contains approximately 500 times more melatonin than the pineal gland. The circulating melatonin absorption has been proven by the increasing plasma melatonin levels from daytime to nighttime, with a significant portion being released without undergoing metabolism into the gut lumen. Daytime blood plasma melatonin levels can be enhanced by consuming melatonin-rich vegetables, while decreases were observed after consuming melatonin-depleted food. The role of intestinal bacteria in impacting the melatonin content of the gut has been addressed but remains an unresolved question. Numerous additional tissues and cells have been recognized as sites for melatonin synthesis, including the human ovaries, testicles, and bone marrow [3].

Several studies have emphasized the remarkable melatonin level decline in neurodegenerative disorders like Alzheimer’s disease and other forms of senile dementia. Through the advanced stages of Alzheimer’s disease, this decline becomes more pronounced, affecting symptoms like sundowning and disrupted sleep. Although there are potential benefits in the earlier stages of using melatonergic treatment, the effectiveness of improving these symptoms are impeded during these late stages due to the decline of melatonin receptors [4].

Research suggests that women with breast cancer exhibit lower melatonin levels compared to those without the disease. Laboratory experiments also indicate that reduced melatonin levels may contribute to the growth of breast cancer cells [5]. Similarly, colorectal cancer patients were found to have lower plasma melatonin levels than healthy individuals, implying a potential association between decreased melatonin levels and an increased risk of colorectal cancer development in humans [6].

In a study demonstrated in healthy rats, melatonin administration established dose-dependent reductions in heart rate, mean arterial pressure, and serotonin release in the hypothalamus and corpus striatum. Notably, the dose–response correlation indicated a more evident effect with higher doses. Melatonin-induced bradycardia that was observed in the rat model was corrected after bilateral vagotomy. Over a five-day melatonin infusion period, a gradual decline in blood pressure, heart rate, and plasma renin concentration was noted, suggesting a sustained impact [7].

Beyond its role in sleep regulation, melatonin administration has been linked with enhanced cardiac vagal tone and parasympathetic nervous system function. It also appears to reduce sympathetic tone, leading to a decline in circulating norepinephrine levels, suggesting potential cardiovascular benefits. In a study published in the American Heart Journal, the administration of 2 mg of melatonin to awake men resulted in decreased heart rate, blood pressure, and increased heart rate variability compared to a placebo group, a trend that was observed in this patient throughout their hospitalization period [8]. Another study involving patients with postural tachycardia syndrome established that melatonin significantly reduced heart rate after two hours compared to a placebo [9].

In a randomized clinical trial studying the heart rate effect of melatonin during submaximal exercise in healthy men, those who ingested 6 mg of melatonin demonstrated significant HR variations compared to the placebo group. The study reported a 6.6% decrease in HR at 10 min into the exercise, declining to 3.6% toward the end. This led to the conclusion that higher doses of melatonin provoke bradycardia during exercise, a finding that may have relevance to the higher dose consumed in the current case. The study speculated a potentially unfavorable effect of Melatonin on cardiovascular function and thermoregulatory control. This effect suggests that the associated decline of free catecholamine production may indicate a subtle impact on physiological responses during physical activity [10].

On the other hand, a study suggested the potential for melatonin to improve the nocturnal reduction in heart rate due to its thermoregulatory and hypnotic effects. This study demonstrated a significant decrease in heart rate for up to three hours following melatonin ingestion compared to temazepam, paralleling the case outlined in this study, where bradycardia manifested one hour after Melatonin ingestion. Moreover, it was noted that the melatonin effect is possibly a direct impact of melatonin from the hypothalamus, its antioxidant properties, or its aortic walls smooth muscles relaxation [11, 12]. Additional studies suggest that supplementing with exogenous melatonin in the evening has the potential to alleviate central nervous system (CNS) side effects, such as sleep disorders linked to the use of beta-blockers. In addition, it may moderate the risk associated with decreased endogenous melatonin synthesis [13].

Despite the comprehensive findings from these studies, this patient in this particular case report displayed marked bradycardia while awake, remaining asymptomatic, and maintaining normal blood pressure, it is crucial to emphasize that the dosage consumed by this patient exceeded the amounts investigated in the above-mentioned studies. Also, it’s worth noting that even under usual circumstances, the baseline heart rate for this patient occasionally drops to the 50s.

## Conclusion

Although melatonin has limited number of documented side effects in humans, melatonin’s significance extends beyond its role in sleep regulation and its potential impacts on cardiovascular health and neurodegenerative disorders. This case report shows a noteworthy association between melatonin and significant bradycardia in previously healthy adults. The actual mechanism through which melatonin impacts cardiovascular function remains unclear, raising questions about whether its effects are due to direct localized action or indirect involvement through thermoregulatory control centers in the hypothalamus. A lack of verified adverse side effects does not indicate their absence. Further research in this field promises to develop many valuable therapeutic interventions to improve our awareness and management of melatonin-related effects on cardiovascular health.
